# Engaging with the private healthcare sector for the control of tuberculosis in India: cost and cost-effectiveness

**DOI:** 10.1136/bmjgh-2021-006114

**Published:** 2021-10-05

**Authors:** Nimalan Arinaminpathy, Arindam Nandi, Shibu Vijayan, Nita Jha, Sreenivas A Nair, Sameer Kumta, Puneet Dewan, Kiran Rade, Bhavin Vadera, Raghuram Rao, Kuldeep S Sachdeva

**Affiliations:** 1 Department of Infectious Disease Epidemiology, Imperial College London, London, London, UK; 2 Population Council, New York, New York, USA; 3 CDDEP, Washington, District of Columbia, USA; 4 PATH Mumbai Office, Mumbai, India; 5 World Health Partners, Patna, India; 6 Stop TB Partnership, Geneva, Switzerland; 7 Bill and Melinda Gates Foundation, India Country Office, New Delhi, India; 8 Bill and Melinda Gates Foundation, Seattle, Washington, USA; 9 World Health Organization Country Office for India, New Delhi, India; 10 USAID India, New Delhi, India; 11 National Tuberculosis Elimination Programme, India Ministry of Health and Family Welfare, New Delhi, India; 12 South-East Asia Office, International Union Against Tuberculosis and Lung Disease, New Delhi, India

**Keywords:** tuberculosis, health economics

## Abstract

**Background:**

The control of tuberculosis (TB) in India is complicated by the presence of a large, disorganised private sector where most patients first seek care. Following pilots in Mumbai and Patna (two major cities in India), an initiative known as the ‘Public–Private Interface Agency’ (PPIA) is now being expanded across the country. We aimed to estimate the cost-effectiveness of scaling up PPIA operations, in line with India’s National Strategic Plan for TB control.

**Methods:**

Focusing on Mumbai and Patna, we collected cost data from implementing organisations in both cities and combined this data with models of TB transmission dynamics. Estimating the cost per disability adjusted life years (DALY) averted between 2014 (the start of PPIA scale-up) and 2025, we assessed cost-effectiveness using two willingness-to-pay approaches: a WHO-CHOICE threshold based on per-capita economic productivity, and a more stringent threshold incorporating opportunity costs in the health system.

**Findings:**

A PPIA scaled up to ultimately reach 50% of privately treated TB patients in Mumbai and Patna would cost, respectively, US$228 (95% uncertainty interval (UI): 159 to 320) per DALY averted and US$564 (95% uncertainty interval (UI): 409 to 775) per DALY averted. In Mumbai, the PPIA would be cost-effective relative to all thresholds considered. In Patna, if focusing on adherence support, rather than on improved diagnosis, the PPIA would be cost-effective relative to all thresholds considered. These differences between sites arise from variations in the burden of drug resistance: among the services of a PPIA, improved diagnosis (including rapid tests with genotypic drug sensitivity testing) has greatest value in settings such as Mumbai, with a high burden of drug-resistant TB.

**Conclusions:**

To accelerate decline in TB incidence, it is critical first to engage effectively with the private sector in India. Mechanisms such as the PPIA offer cost-effective ways of doing so, particularly when tailored to local settings.

**Key questions**

**What is already known?**

- In order to accelerate current declines in tuberculosis burden in India, it is critical to engage effectively with the country’s vast private healthcare sector.
- Previous work has examined the potential impact of such measures on incidence and mortality, but it remains important also to estimate their cost-effectiveness.

**What are the new findings?**

- Using cost data from Mumbai and Patna, two major cities in India, the new findings show that scaling up private sector engagement would be cost-effective in both settings.
- However, to be cost-effective in settings with low burden of drug resistance such as Patna, the intervention should focus on improving treatment outcomes, rather than diagnosis, in the private sector.

**What do the new finding imply?**

- Private sector engagement in Indian cities will be cost-effective even with respect to the most stringent criteria, particularly when tailored to local settings.

## Introduction

In India, the country with the world’s largest tuberculosis (TB) burden,^1^ a major challenge is that many patients continue to be treated in the private healthcare sector,^2–4^ where the quality of diagnosis and treatment support is often poor,^4–6^ and notification to public health authorities is limited.^7^ Engaging effectively with this sector forms a critical foundation of India’s TB response.^8^ From 2013 to 2017, India’s Central TB Division led pilot projects in Mumbai and Patna, two major Indian cities, that showed how private providers could be effectively engaged through Public–Private Interface Agencies (PPIAs). In brief, this mechanism: (i) makes available high-quality diagnostic tests to private providers through incentives, subsidies and eventually free of cost, (ii) provides free TB drugs and adherence support mechanisms to TB patients to maximise treatment completion and (iii) facilitates reporting of TB patients to India’s National Tuberculosis Elimination Programme. Following these pilots, in recent years, private sector engagement has seen massive expansion across the country, accompanied by an increase in the Government of India’s pledged budget for TB, as well as support from the World Bank and the Global Fund.^9^ Consequently, the private sector contribution to TB notifications grew from 7% in 2014 to 28% in 2019.^10^ While there remains much ground to be covered in achieving comprehensive coverage of private providers in India, these developments and ongoing efforts represent crucial steps in this direction.

In previous work, we developed a mathematical model of TB transmission to estimate the potential impact of private sector engagement on TB incidence and mortality, when taken to scale in urban slums such as in Mumbai and Patna.^11^ That work illustrated in particular that private sector engagement may not lead to large reductions in TB incidence when acting alone, but would be a critical foundation for a broad TB response. Here, we built on this earlier modelling, to estimate the cost-effectiveness of these efforts. We aimed to address the questions: how cost-effective would a PPIA be, when taken to scale in urban settings in India? In a given setting, which components of a PPIA (eg, among improved diagnosis and treatment interventions) matter most, for efficiency in improving health outcomes? Concentrating on Mumbai and Patna, we collected cost data collected directly from PPIA activities in each of these settings. By incorporating these costs into the previously published transmission modelling framework,^11^ we captured the health gains as a result of improving TB outcomes, as well as the impact of reducing TB transmission.

## Methods

### Overview of PPIA operations

Although highly diverse, the private healthcare sector in India can broadly be divided in three categories: formally qualified (FQ) providers who are qualified in allopathic medicine; less-than-fully-qualified (LTFQ) providers who have no such qualification (including ‘informal’ healthcare providers); and chemists. Evidence suggests that the latter typically do not offer TB treatment over-the-counter.^12^ Consistent with PPIA operations, we consider interventions aimed at engaging FQs and LTFQs only.

Providers in both Mumbai and Patna have been sensitised and engaged through visits by field officers and events such as seminars and training workshops. To encourage better TB diagnosis, the PPIA offered patient subsidies and eventually free diagnostic tests (rapid molecular tests, chest radiographs and sputum smear microscopy) and support in sputum sample collection through field staff. To support treatment outcomes, the PPIA provided free TB drugs, as well as linkage to a call centre for adherence monitoring and support. In addition, engaged providers in Patna were provided with incentives for symptomatics tested, TB patients diagnosed and TB patients completing treatment. In both sites, patients found to have drug-resistant (DR) TB were referred to the public sector for treatment.

### Transmission model

**Figure 1:**
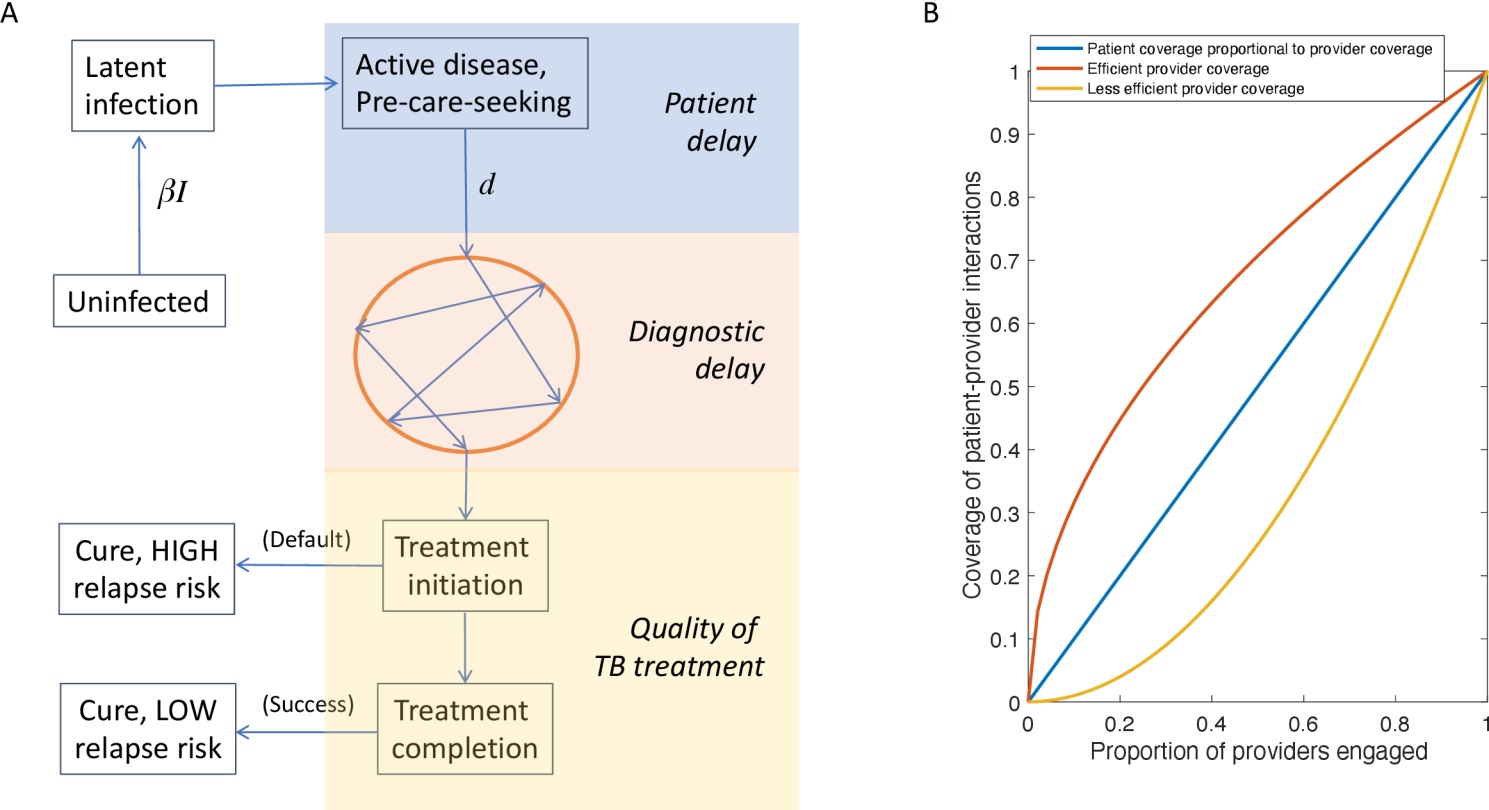
Schematic illustration of the model structure. (A) Overview of the compartmental model structure, described in detail in ref.^11^ The circle denotes the interval from a patient’s first presentation to care until ultimate TB diagnosis, during which they may visit several different providers in both the public and private sectors. By improving diagnosis in the private sector, a PPIA aims to remove patients from this loop as rapidly as possible. Additionally, by providing free drugs and adherence support in the private sector, a PPIA aims to minimise the risk of long-term recurrence (bottom left compartment). (B) A simple approach for linking PPIA ‘provider coverage’ (the number of providers recruited of a given type, for example, FQ) with ‘market share’ (the proportion of provider–patient interactions captured by a PPIA). The former is relevant for costing, while the latter is relevant for the transmission model illustrated in panel A. Curves arise from the formula: Market share = (Provider coverage)*^k^*, for a given parameter *k*. Ideally a PPIA would first recruit the highest-caseload providers, to capture a disproportionate amount of patient–provider interactions (*k*=0.5, red curve). However, we also allow conservatively for less efficient provider recruitment (*k*=2, yellow curve). In propagating uncertainty through Bayesian melding, we allow the control parameter *k* to vary uniformly between 0.5 and 2. FQ, formally qualified; PPIA, Public–Private Interface Agencies; TB, tuberculosis.

A PPIA can avert disease and mortality through reducing opportunities for transmission, as well as through improved outcomes for TB patients: it is important to capture these potential ‘indirect’ effects in any approach estimating potential cost-effectiveness.^13^ The mathematical framework used to capture TB transmission is illustrated schematically in figure 1A and described in detail elsewhere,^11^ and with further technical details provided in the supporting information. In brief, the model captures TB patient pathways in Mumbai and Patna, and epidemiological data for TB relevant to urban slums in Mumbai and Patna (table 1). On the basis of these inputs, the model projects the potential impact of a PPIA at a given scale, on TB transmission. The model incorporates the acquisition and transmission of rifampicin resistance and multidrug resistance, together referred to here as ‘DR’ TB.

**Table 1:** Epidemiological indicators used in the calibration

Indicator	Setting	Value	Source
Annual risk of TB infection as of 2015	Mumbai and Patna	2%–3%	Gopi (2008)^39^
Prevalence as of 2015	Mumbai and Patna	388 per 100 000 (233–543)	Baskaran (2015)^40^
Percent of incident TB that is drug-resistant	Mumbai	12% (8–16)	Assumption
	Patna	4% (3–5)	Assumption

TB, tuberculosis.

We divided private providers into FQ, LTFQ and chemists. The model captures the ‘diagnostic delay’ arising from patient movement between these different types of providers, using data from patient pathway surveys from both Mumbai and Patna.^14^ Parameters relating to the quality of TB care are summarised in online supplemental table S1. In brief, we assumed that all engaged providers diagnose a higher proportion of TB patients than unengaged providers;^8 15^ FQ providers, having access to rapid molecular tests, are also able to recognise DR-TB at the point of TB diagnosis. With increased diagnostic accuracy among engaged private providers, the model captures the potential effect of a PPIA in reducing the diagnostic delay.

10.1136/bmjgh-2021-006114.supp1 Supplementary data

To model the impact of treatment adherence interventions, we assumed that patients treated under private providers have lower rates of treatment completion than in the public sector, and that ‘engagement’ with the PPIA would raise these rates to match those of the public sector. We assumed conservatively that any patient with drug-susceptible (DS)-TB initiating treatment is rendered non-infectious: those interrupting treatment are subject to a higher risk of recurrent TB in the 24 months following treatment, than those completing treatment. In the absence of systematic evidence for these risks in the context of private sector engagement, we drew from the relevant literature.^16^

When simulating the potential impact of a PPIA at a given scale, as the model is based on a longitudinal perspective of patient pathways, the measure of scale relevant to the model is that of ‘market share’, that is, the proportion of patient–provider interactions captured by a PPIA. However, from the costing perspective, the more relevant measure of scale is the ‘provider coverage’ of a PPIA, or the number of providers engaged. Market share is not necessarily proportional to provider coverage, owing to wide heterogeneity in the number of TB patients that providers manage, even among FQ or LTFQ providers. In integrating costing and transmission models, we therefore sought to link market share to provider coverage in a simple way, as illustrated by figure 1B.

### Costing

We estimated the provider cost of PPIA separately in Mumbai and Patna using an ingredient-based approach. The broad cost categories were: (i) cost of engaging healthcare providers with the PPIA network, (ii) cost of TB diagnostic tests and patient follow-up and (iii) treatment cost. The PPIA programme was implemented and managed by a different non-governmental organisation (NGO) in each location at the time of data collection.

In Mumbai, we collected data on PPIA output and cost for the period September 2014 to May 2015. Human resources for the Mumbai programme included field staff members of the main NGO and its affiliate NGOs, who were engaged primarily with FQ, LTFQ and diagnostics providers. Two managerial positions at the main NGO were involved in programme management and supervision of field teams. We interviewed the managers to obtain approximate distributions of human resources across various programme activities. We estimated the cost of engaging one FQ or LTFQ provider by combining two cost components—the value of full-time equivalent (FTE) cost of staff involved in engaging providers, and the per capita cost of provider orientation workshops conducted by the NGO.

Diagnostic tests (X-ray and rapid molecular tests) for those presumed to have TB in the PPIA were conducted by a network of third-party, private laboratories in Mumbai. These providers were compensated by the programme through a voucher system. We included the average value of the vouchers and the FTE cost of human resources engaged in overseeing TB diagnostics to estimate the PPIA cost for each test.

We collected cost data from the PPIA implementing NGO in Patna during March 2015. The Patna PPIA model differed from the Mumbai in two main aspects which affected our cost estimates. First, while sputum smear microscopy and chest X-ray under PPIA were conducted by third-party providers (through a voucher system) in Patna, GeneXpert tests were primarily (approximately 90%) conducted at the PPIA implementing NGO’s own laboratory. Second, in addition to programmatic costs, the Patna PPIA provided cash incentives ranging between $1.5 and $7 on a per case basis to FQ and LTFQ providers, patients.

We calculated the cost of engaging each FQ and LTFQ provider in Patna by combining the programme cost per capita and the value of cash incentive provided to each provider. For GeneXpert tests conducted at the NGO facility, we first estimated the annual value of capital (test equipment and installation) at the rate of 15% depreciation per year. Then, we added the annual cost of maintenance for the equipment and the annual FTE values of one supervisor (partial time commitment) and one technician (full time commitment) conducting the tests. We divided the total cost by the estimated number of GeneXpert tests that the facility could conduct in a year to obtain the cost of each test. For the small share of GeneXpert tests conducted in third-party laboratories, we considered the value of voucher payments to the providers as the cost.

FTE values of no other NGO human resources working on PPIA in Patna were available. In Mumbai, the FTE value of non-laboratory staff involved in managing and monitoring GeneXpert tests was approximately $25 per test. We assumed that this component would be similar in Patna and added it to the per test cost discussed above. Thus, our estimate of per GeneXpert test cost included the values of capital, maintenance and laboratory staff (or vouchers), along with the value of non-laboratory staff.

### Model calibration and uncertainty

Table 1 shows the TB burden indicators used to calibrate epidemiological parameters in the model. This includes annual risk of TB infection and TB prevalence typical of urban slums in India, as well as allowing for a higher burden of DR-TB in Mumbai than in Patna. With a lack of public data from drug resistance surveys specific to Mumbai, there is some uncertainty on the true burden of DR-TB there, for example with previous, facility-based studies suggesting 11%–67% among previously treated patients.^17^ We assumed a burden among all incident TB towards the lower end of this range, of 12%: as discussed below, this makes our analysis conservative with respect to the cost-effectiveness of the intervention in Mumbai. For Patna, we assumed a comparable burden of drug resistance as on the country level.^1^

All inputs are subject to uncertainty, as are the patient pathway parameters described above. To capture systematically the implications of this combined uncertainty for the cost-effectiveness of a PPIA at scale (ie, to ‘propagate’ uncertainty from model inputs to model outputs), we used Bayesian melding, an approach first developed to inform burden estimates for HIV.^18^ In addition to the model parameters, we also allowed for 25% error in the unit costs used to estimate overall spending.

### Cost-effectiveness analysis

PPIA operations began in Mumbai and Patna in 2014, and were scaled up over subsequent years. The purpose of our current analysis is not to assess the cost-effectiveness of ongoing efforts, but rather to examine a range of possible scenarios for PPIA deployment, including varying levels of provider coverage in both settings, as well as prioritising either diagnosis or treatment, rather than combining these services. This approach allows us to draw lessons from the current analysis, for optimal PPIA approaches in other settings. We therefore simulated PPIA interventions from 2014 to 2025, assuming a linear increase in provider recruitment over 3 years from 2014 to 2017 and, as a comparator, assuming public and private sector TB care both to continue indefinitely at their pre-2014 levels. We measured health impact using disability adjusted life years (DALYs), which account for the disability due to TB disease as well as the years of life lost due to premature, TB-related mortality. We calculated the incremental spend and DALYs averted from 2014 to 2025. We thus computed the incremental cost-effectiveness ratio (ICER) as the ratio of incremental cost to incremental DALYs averted. We incorporated 3% annual discounting in both impact and cost estimates, for example as in refs.^19 20^

To assess cost-effectiveness, we used two approaches for ‘willingness-to-pay’ thresholds. First, we followed an approach recommended by WHO-CHOICE, of evaluating GDP per capita (g), in each city: where ICER§amp;lt;3g, the intervention is considered ‘cost-effective’. Where ICER§amp;lt;g, the intervention is considered ‘highly cost-effective’. Although these thresholds have the advantage of simplicity, transparency and wide adoption, recent work has proposed new, country-specific thresholds incorporating more systematic analysis of opportunity costs of intervention, as shaped by the efficiency of the healthcare system in different settings.^21^ In India, these thresholds are more stringent than GDP per capita; we therefore also compared model-based ICER estimates against these alternative thresholds. All willingness-to-pay thresholds are summarised in table 2.

**Table 2:** WTP thresholds used to assess cost-effectiveness

Type of threshold		Mumbai	Patna
WHO-CHOICE (based on GNP per capita)	Highly cost-effective	$1500	$530
	Cost-effective	$4500	$1590
More stringent WTP*		$290	$290

**Figure 2:**
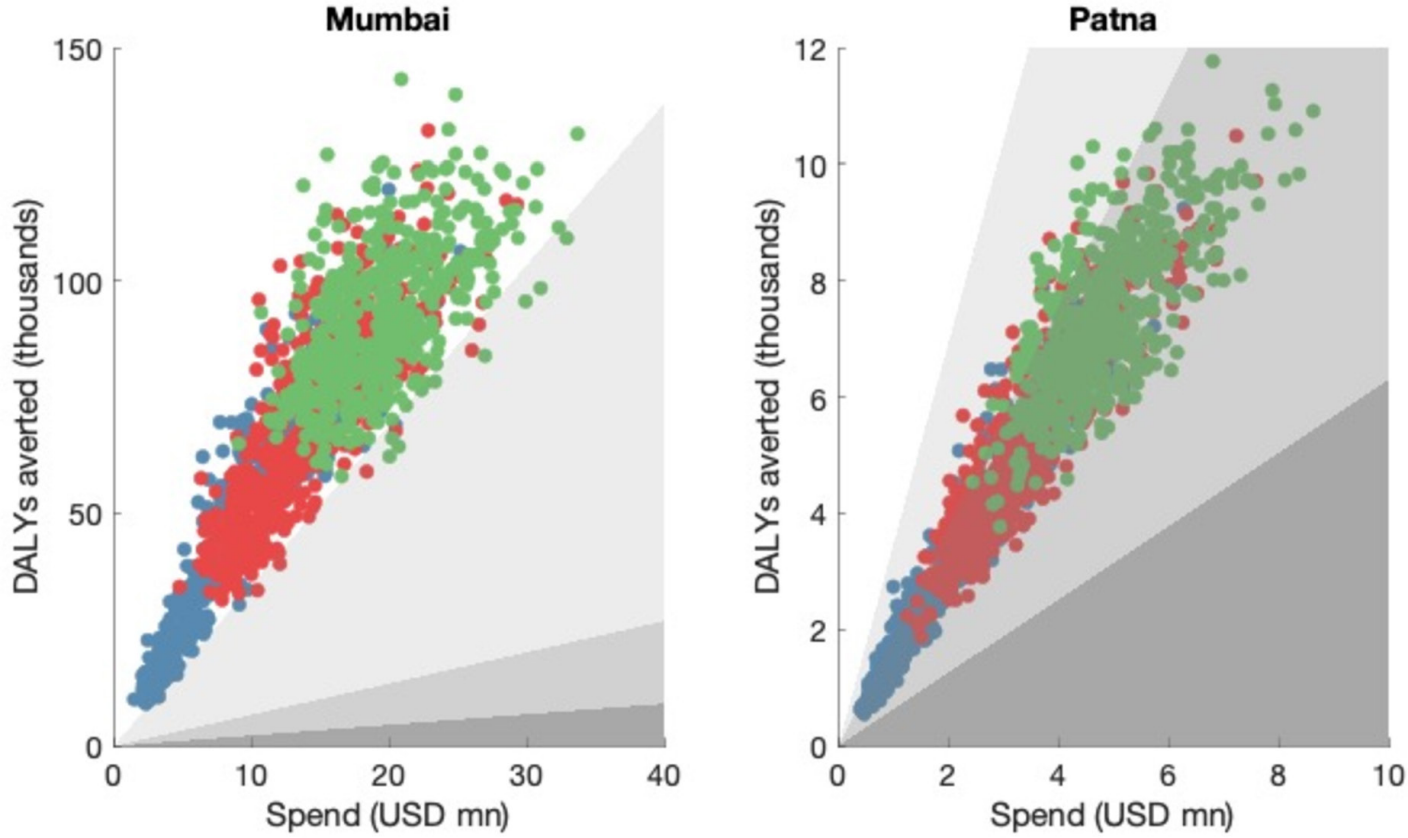
Cost-effectiveness plane of PPIAs operating at scale. Different ‘dots’ of a given colour arise from uncertainty in model inputs, and in the unit costs used. Blue, red and green dots represent provider coverage scenarios of 25%, 50% and 75%, respectively. Shaded regions show willingness-to-pay thresholds listed in table 2, as follows: points in the white region are cost-effective with respect to all thresholds listed in table 2. Points in the lightest grey region are ‘highly cost-effective’ with respect to the WHO-CHOICE threshold, but not cost-effective with respect to the ‘stringent’ threshold. Points in the middle grey region are cost-effective with respect to WHO-CHOICE, and the dark grey region shows parameters that are not cost-effective under any of the thresholds listed in table 2. Both horizontal and vertical axes correspond to cumulative totals over the period 2017 to 2025.

**Figure 3:**
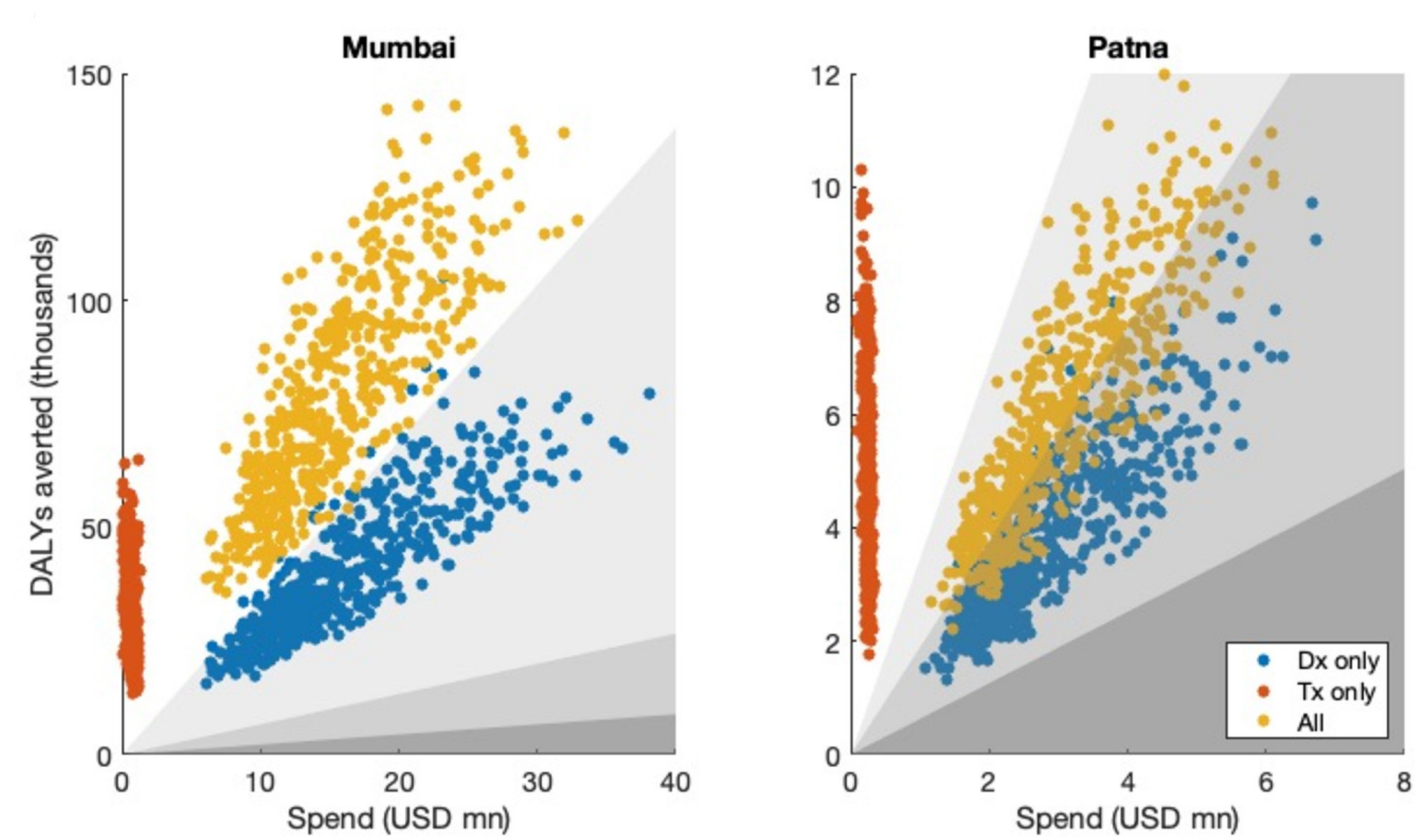
Cost-effectiveness planes showing different prioritisations of PPIA activities. Results show the case of 50% engagement of private providers in both cities. Points in blue show a scenario where the PPIA focuses on improving diagnostic quality among private providers, without addressing patient treatment outcomes. Points in red show the converse scenario where the PPIA focuses on improving patient treatment outcomes, without addressing improved diagnostics. Points in yellow show combined efforts to improve diagnostic and treatment quality. Shaded regions show willingness-to-pay thresholds listed in table 2, as follows: points in the white region are cost-effective with respect to all thresholds listed in table 2. Points in the lightest grey region are ‘highly cost-effective’ with respect to the WHO-CHOICE threshold, but not cost-effective with respect to the ‘stringent’ threshold. Points in the middle grey region are cost-effective with respect to WHO-CHOICE, and points in the dark grey area satisfy none of the three thresholds for cost-effectiveness. In general, the greater the angle between any given point and the positive X-axis, the more favourable it is in cost-effectiveness terms. In both settings, therefore, PPIAs focusing only on treatment outcomes will be most cost-effective (red points) but in Mumbai, such a strategy would substantially compromise overall health impact, by neglecting diagnostics (comparison of red and yellow points). DALY, disability adjusted life years; PPIA, Public–Private Interface Agencies.

The table shows different threshold for cost-effectiveness, used in figures 2 and 3.

*This estimate is drawn from recently published analysis,^21^ for which only country-level estimates are available. The study used different approaches to propose three different WTP thresholds for India; for simplicity in the present study, we adopt the most stringent of these.

WTP, willingness-to-pay.

## Results

Table 3 shows the unit cost estimates for PPIAs in both Mumbai and Patna. Using this data, we simulated the potential cost and impact of a PPIA in both Mumbai and Patna, subject to uncertainty in epidemiological estimates, care seeking parameters and unit costs. Figure 2 shows the resulting cost-effectiveness planes for both Mumbai and Patna, for different levels of provider coverage, ranging from 25% to 75%. In Mumbai, a PPIA would meet all three criteria for cost-effectiveness, including the most stringent criterion incorporating opportunity costs in the health system. However in Patna, a PPIA may or may not be cost-effective, depending on the choice of willingness-to-pay threshold: compared with the WHO-CHOICE threshold, a PPIA would be cost-effective, that is, with each DALY averted costing less than three times GNP per capita. However, it would not be cost-effective compared with the most stringent threshold incorporating opportunity costs.

**Table 3:** Unit costs used in the analysis

Cost component		Unit	Mumbai	Patna
Provider engagement	FQ	Per provider engaged	108	53
	LTFQ		108*	130
Diagnosis	FQ (Xpert)	Per patient tested	44	27
	LTFQ (Smear and X-ray)		4	4
Treatment	First-line	Per patient-month of treatment	8	7
	Second-line		100	100

As described in the main text, costs were developed through data on PPIA operating costs and interviews with implementing partners. See methods for more information on cost calculations. All costs are given in US dollars, and subject to variation within ±25% in the uncertainty analysis. See online supplemental table S2 in the supporting information for a further breakdown of these costs. Note that the PPIAs in Mumbai and Patna differed in several important respects, including the service delivery model and incentive structures employe, the provider profile and provider behaviour on test preferences, and health infrastructure availability (drugs and diagnosis). Consequently, any direct comparison between Mumbai and Patna costs should be performed with caution.

*A breakdown of provider engagement costs by provider type was not available in Mumbai; we therefore assumed the same costs per FQ and LTFQ provider. In practice, the cost of LTFQ engagement is generally expected to be lower than the cost stated here for FQs; our analysis is thus conservative by tending to overestimate the overall cost of private sector engagement.

FQ, formally qualified; LTFQ, less-than-fully-qualified; PPIA, Public–Private Interface Agencies.

These results are for the scenario of a PPIA combining efforts to promote both diagnostic and treatment quality among private providers. To better understand the contribution of these separate functions, we next examined whether a ‘reduced’ PPIA, focusing on either diagnosis or treatment alone, could achieve similar impact as the full PPIA but more efficiently (ie, with reduced cost). Figure 3 shows results for both Mumbai and Patna, in the illustrative example of a PPIA operating at 50% provider coverage in both settings. These results, together with estimates for ICERs summarised in table 4, illustrate that a treatment-focused PPIA appears to offer the greatest value in both settings, with less cost per DALY averted than the ‘combined’ PPIA. Notably however, in Mumbai, limiting PPIA activities to treatment could significantly compromise its impact: the inclusion of diagnostic support more than doubles the health impact, from 26 000 to 60 000 DALYs averted. By contrast in Patna, a focus on treatment services could render a PPIA cost-effective even under the most stringent of willingness-to-pay thresholds, with little reduction in overall impact.

**Table 4:** Summary of simulated cost and impact under different PPIA scenarios

Site	PPIA scenario	DALYs averted (thousands)	Incremental spend (US$ millions)	US$ per DALY averted
Mumbai	**Dx only**	29.4 (15–56.4)	12.8 (6.64–26.2)	441 (319–601)
	**Tx only**	24.4 (12.6–47.2)	0.732 (0.153–1.16)	30.5 (3.46–79.7)
	**Both**	54.8 (30.3–95.5)	12.4 (6.76–24.1)	228 (159–320)
Patna	**Dx only**	2.87 (1.41–5.99)	2.35 (1.25–4.78)	803 (566–1120)
	**Tx only**	3.49 (1.82–6.81)	0.255 (0.169–0.315)	72.6 (29.6–157)
	**Both**	4.27 (2.24–8.44)	2.46 (1.34–4.77)	564 (409–775)

As described in the main text, ‘Tx only’ refers to a reduced PPIA that focuses efforts and spending in adherence support, while ‘Dx only’ focuses instead on quality of TB diagnosis. All estimates incorporate 3% annual discounting in both DALYs averted and incremental spend. Numbers are given under the assumption of 50% provider coverage. See also figure 3 for a visualisation of these results.

DALY, disability adjusted life years; PPIA, Public–Private Interface Agencies.

**Figure 4:**
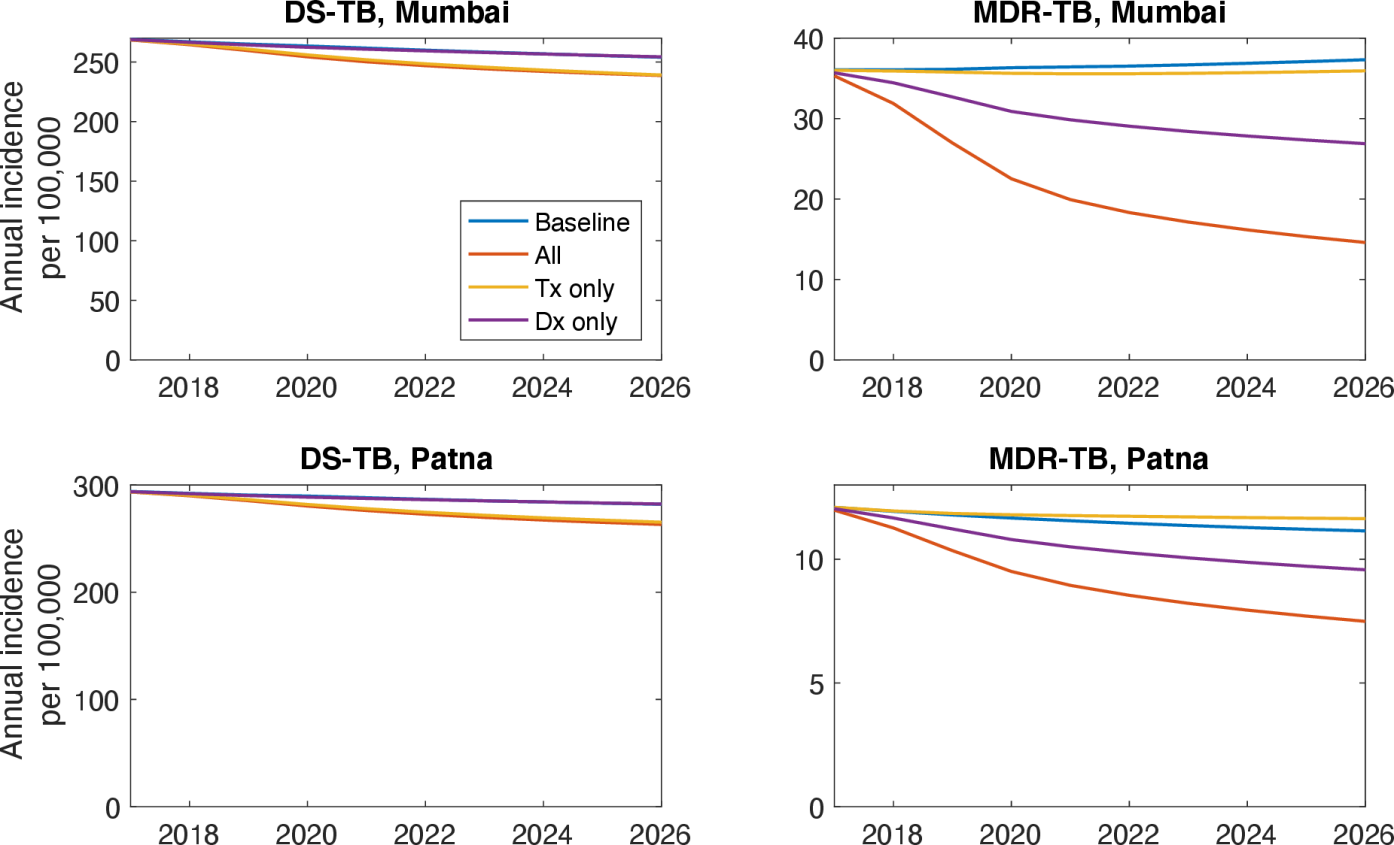
Illustrative impact of the different ‘arms’ of a PPIA. Shown are epidemic curves under different scenarios, assuming 50% coverage, for Mumbai (top row) and Patna (lower row). For DS-TB (left-hand panels), a PPIA concentrating on treatment has essentially the same effect as a ‘full’ PPIA that addresses both treatment and diagnosis (yellow and orange curves, overlaid). A PPIA concentrating on diagnosis, however, has little impact on incidence (blue and purple curves, overlaid). These dynamics illustrate that, for DS-TB, the incidence impact of a PPIA arises largely from controlling recurrence rates through treatment completion, rather than by reducing the delay to diagnosis. For DR-TB (right-hand panels), however, diagnosis plays a stronger role in incidence reduction than treatment: this is because of the low current rates of drug sensitivity testing at the point of TB diagnosis, that could be addressed by providing drug-susceptibility testing to patients cared for by the private sector. DS-TB, drug-susceptible tuberculosis; PPIA, Public–Private Interface Agencies.

Figure 4 illustrates the epidemiological behaviour underlying these results, showing DS and drug-resistant (DR-) TB separately. These results illustrate that, for DS-TB, a PPIA’s impact on incidence comes largely from treatment support, in particular the reduced recurrence risk arising from treatment adherence. By contrast, improved diagnostics have little impact on DS-TB incidence. However, for DR-TB, the opposite is true: early recognition of DR-TB, through the availability of rapid molecular tests in the private sector, would have a strong impact on incidence, substantially more so than improved linkage to second-line treatment. Overall, these results illustrate that a major reason for the difference in cost-effectiveness between Mumbai and Patna is the burden of DR-TB in these settings: in general, settings with a higher burden of DR-TB would be expected to see greater incremental value of improved diagnostics in the private sector.

## Discussion

Effective engagement with India’s vast and fragmented private healthcare system will offer important opportunities to extend high-quality care to all TB patients, regardless of where they seek care. Although the importance of such engagement has been recognised for some time,^22 23^ it is only in recent years that the PPIA mechanism has emerged as an effective, scalable solution in India.^8^ Here, by incorporating programmatic costs into transmission modelling, our results show how these measures can be cost-effective when taken to scale. Our findings may have relevance to other settings where the private sector plays an important role in TB care, especially in South and South-East Asia; at a time when budget pledges do not always translate to allocated funding in practice,^1^ this analysis highlights the important health gains that could be achieved from mobilising such funding.

Notably, our results highlight the value of investment to support treatment adherence in any setting: given the comparatively low cost of such an intervention, as well as its impact on future recurrence and thus TB incidence, improving treatment adherence alone would be cost-effective in both of the settings explored here, even under the most stringent WTP thresholds (table 4). For adherence support mechanisms we have focused on free, government-supplied drugs, as well as patient counselling and linkage to a call-centre for ongoing support during the course of treatment. However, other initiatives may also act to promote treatment adherence, including ‘Nikshay Poshan Yojana’, a welfare scheme in India that offers monthly financial support to patients on TB treatment,^24^ whether in the public or private sector. Future modelling analysis could incorporate the effects of this scheme, as more data become available on its impact. Overall, however, our analysis highlights the importance of continued investment in these and other initiatives to support treatment adherence, as important components of private sector engagement.

Improving diagnosis is another major component of any PPIA, and our analysis highlights how efforts in this direction may need to be tailored to local settings. In particular, access to molecular diagnostics capable of rapid DST would be particularly important in settings that face a high burden of DR-TB. In a city such as Mumbai, such diagnostic tools facilitate early recognition of DR-TB among private providers, leading to important reductions in the incidence of DR-TB, as well as substantial, long-term cost savings in the management of DR-TB. Thus, our results suggest that combining diagnostic and treatment completion efforts would be either cost-effective or highly cost-effective, depending on the choice of WTP threshold (table 4). By contrast, in settings such as Patna having a comparable overall burden of TB but comparatively low rates of drug resistance, a focus on treatment completion rather than diagnosis would be cost-effective regardless of the choice of WTP threshold (figure 3).

Our work adds to other modelling studies that have also addressed the cost-effectiveness of private sector engagement in India and the South-East Asian Region; Menzies *et al*^25^ combined different transmission models to estimate the potential impact of different interventions in three countries, including India, showing that—from the health systems perspective—efforts to improve diagnosis and treatment throughout the healthcare system (including the private sector) were likely to be cost-effective. Likewise, Bhatia *et al*^26^ built on previous modelling analysis^27^ to estimate the resource needs for scaling up TB interventions in the WHO South-Eastern Asian Region. This analysis estimated, for example, that India would require an additional US$ 0.2 per capita, to sustain scaled-up private sector engagement between 2017 and 2030. However, both of these studies were informed by data from older ‘public–private mix’ initiatives in India.^28^ Our analysis adds to this evidence basis by providing more up-to-date estimations of cost-effectiveness, with primary cost data drawn from the new PPIA mechanism being adopted across the country.

As PPIAs continue to be adopted across the country, there are several ways in which our analysis could be further refined. Our primary cost data reflect differences in the implementation of PPIA pilots with, for example, Patna seeing more extensive use of cash-based incentives than Mumbai, partly explaining the difference in intervention costs for provider recruitment between the two settings (online supplemental table S2). As these programmes mature from pilots to country-level implementation, and as they are further optimised in the process, we would only expect their cost-effectiveness to improve over time. Moreover, we note that our estimates of impact are driven partly by assumptions for the uptake of services such as adherence support. As PPIAs continue to be adopted, it will be important to collect data on the actual uptake of these services. Because it is often infeasible to measure changes in incidence directly, it is likely that mathematical modelling will remain a useful tool for projecting these changes in future. Nonetheless, these data on ‘real world’ uptake of PPIA services will be in refining modelling of the impact of PPIA interventions, as they are implemented in reality.

In certain respects, our work is conservative in its estimated impact and cost-effectiveness of PPIAs. For example, our cost-effectiveness analysis is limited to the programme perspective, and does not incorporate the potential for important benefits to patient costs. Coping with TB can be an important cause of catastrophic health expenditure;^29–31^ by helping to shorten the diagnostic pathway and providing free TB treatment, a PPIA can have important impact in reducing these patient costs. We would expect any future analysis, incorporating a fully societal perspective, to show greater cost-effectiveness than in the present analysis. Additionally, our analysis does not capture the potential impact of a PPIA in facilitating ‘downstream’ interventions, for example, preventive therapy among household contacts of diagnosed TB patients. Previous modelling analysis has illustrated the additional impact that could be achieved, if preventive therapy among household contacts is implemented among patients being managed in the private, as well as public, sector.^32^ We would therefore expect inclusion of these and other ‘downstream’ interventions to further enhance the epidemiological impact of a PPIA. Finally, given the lack of systematic data specific to Mumbai, we have taken a deliberately conservative approach for the proportion of TB that is drug-resistant (table 1). If, as is likely, the true burden of drug-resistance in Mumbai is greater than that assumed here, we would expect the cost-effectiveness ratio to be more still favourable than estimated in table 4.

In general, cost-effectiveness considerations are often intended only as a guide for policymakers, with the expectation that they should be combined with other, locally relevant factors such as affordability and implementation.^33^ Moreover, estimating appropriate willingness-to-pay thresholds in different settings is a challenging task, based—for example—on still-debated questions of what constitutes ‘value’ in health, and how such value is affected by resource constraints and other local factors.^34^ Perhaps unsurprisingly in light of these uncertainties, different willingness-to-pay thresholds have emerged. While a ‘conventional’ approach based on GNP per capita has the benefit of transparency and simplicity, recent work proposes new thresholds, taking account of opportunity costs, that are markedly more stringent for India.^21^ To address this uncertainty in the present work, we have simply presented results with respect to both types of threshold. Notably, as described above, our work shows that PPIAs can be cost-effective despite these uncertainties.

As with any modelling approach, our work has limitations to note. First, in the absence of systematic evidence for the potential impact of a PPIA on the risk of recurrence, we have instead drawn from the literature to quantify this risk,^16^ and how it may be reduced with improved treatment completion. At the same time, emerging pill-in-hand adherence technologies are offering new opportunities for adherence support at low cost, such as 99DOTS^35^ as is the Nikshay Poshan Yojana scheme described above. Longitudinal follow-up of TB patients managed by such systems will provide additional valuable evidence in support of future cost-effectiveness assessments. Second, we have had to make assumptions for how to relate provider coverage to ‘market share’ (figure 1B): this relationship is a pivotal step in relating spending to intervention scale, and thus to impact. One approach is to compare drug sales data in the private sector with patient treatment in the public sector: indeed, ongoing data collection for the PPIAs in Mumbai and Patna suggest that both initiatives have captured patients in greater proportion than providers, qualitatively consistent with the red curve in figure 1B. While we have allowed equally for a more conservative scenario in the present work (ie, a less-than-proportionate scaling), future work would benefit from more direct, systematic evidence for the relationship between provider coverage and market share. Third, although our analysis helps to identify what PPIA services might take priority in a given setting, it does not address how best to implement these services. Implementation challenges are likely to vary across such a large and diverse country as India, highlighting the need for locally tailored approaches to optimising PPIA services. Recent findings highlighted the specific conditions in Mumbai,^36^ and similar learnings from other settings in India will be helpful as PPIA activities are taken to scale. Fourth, as with any modelling approach, we have made several simplifications: for simplicity, we did not model paediatric or extrapulmonary TB, focusing instead on infectious forms of TB. We have ignored the difference between smear-positive and smear-negative TB, instead taking an average transmission potential. On the cost side, we have not included one-off costs such as the investment in setting up new call-centre facilities, or the information technology for generating vouchers for diagnosis, focusing instead on the recurring costs that would be incurred over the course of PPIA operations. Finally, this modelling analysis does not address the impact of the ongoing COVID-19 pandemic, and thus ignores disruptions to TB services arising from lockdowns and other pandemic response measures. The adverse effects of these disruptions in TB incidence and mortality can be substantial, and long-lasting.^37^ While there is evidence that the private sector has been affected at least as badly as the public sector,^38^ increased coordination between these sectors is likely to play a key role in bringing TB services back on track as rapidly as possible.

In conclusion, coordinating TB care across India’s vast and complex healthcare system will form a critical foundation for the future of TB control in India. Especially in light of India’s recent, far-reaching National Strategic Plan for TB, such cost-effective measures will go a long way towards alleviating the burden of TB, both in India and elsewhere.

## Data Availability

All data relevant to the study are included in the article or uploaded as supplementary information.
