# Supplementary material for: Engaging with the private healthcare sector for the control of tuberculosis in India: cost and cost-effectiveness
Source: BMJ Glob Health. 2021 Oct 5;6(10):e006114. doi: 10.1136/bmjgh-2021-006114 (PMC8493898; doi:10.1136/bmjgh-2021-006114)
Supplement: Supplementary data [file bmjgh-2021-006114supp001.pdf]

Engaging with the private healthcare sector for the control of tuberculosis in India: Cost and cost-effectiveness

Additional supporting information

1. Model equations

For all model equations given below, table S1 lists definitions of state variables. Here we provide an overview of model equations. For further description of the model, including parameter values; details on patient pathways; and model calibration, see Arinaminpathy et al (2019), Scientific Reports, "Modelling the impact of effective private provider engagement on tuberculosis control in urban India."

Uninfected (U):

$$\frac{dU}{dt} = b - U\sum_{s} \lambda_s - \mu U$$

where b is the birth rate; λ_s is the force-of-infection associated with drug resistance s (given below); and μ is the per-capita, background mortality hazard.

Latent TB infection with drug resistance status s (L_s):

$$\frac{dL_s}{dt} = (1-f)\lambda_s\left[U + \sum_{s}\left(L_s + R_s^{(hi)} + R_s^{(lo)}\right)\right] - (g+\mu)L_s$$

where f is the proportion of infections that are 'fast' progressors; and g is the rate of breakdown to active TB disease.

Active TB disease with drug resistance status s (I_s):

$$\frac{dI_s}{dt} = f\lambda_s\left[U + \sum_{s}\left(L_s + R_s^{(hi)} + R_s^{(lo)}\right)\right] + gL_s + \rho^{(hi)}R_s^{(hi)} + \rho^{(lo)}R_s^{(lo)} - (c + \sigma + \mu_{TB})I_s$$

where $\rho^{(hi)}, \rho^{(lo)}$ are per-capita rates of relapse (detailed below); c is the per-capita rate of careseeking amongst those with active TB; σ is the per-capita rate of self-cure; and μ_{TB} is the per-capita hazard of mortality from TB.

Upon careseeking, visited provider type r and awaiting diagnosis (D_{rs}):

$$\frac{dD_{rs}}{dt} = cp_rI_s + \gamma p_r B_s - (d_r + h_r + \sigma + \mu_{TB})D_{rs}$$

where p_r is the probability of visiting provider type r; γ is the rate of repeat careseeking amongst those who have temporarily dropped out of careseeking; and d_r is the rate of offering a diagnosis.

Diagnosed and on first-line TB treatment (F_{rs}):

$$\frac{dF_{rs}}{dt} = \begin{cases} d_r u_r D_{r,0} - \left(\tau^{(FL)} + \delta_r + \alpha + \sigma + \mu\right) F_{r,0} & r = 0 \\ d_r u_r (1 - v_r) D_{r,1} + \alpha F_{r,0} - \left(\tau^{(FL)} + \delta_r + \sigma + \mu_{TB}\right) F_{r,1} & r = 1 \end{cases}$$

where u_r is the probability of offering a correct TB diagnosis and beginning treatment; $\tau^{(FL)}$ is the rate of completion of first-line TB treatment; δ_r is the rate of loss-to-followup from treatment; α is the rate of DR-TB acquisition while on treatment; and v_r is the proportion of individuals with DR-TB who are initiated on appropriate treatment as a result of their drug-resistance status being properly recognised.

Diagnosed and on second-line TB treatment (DR-TB only, $S_{r,1}$):

$$\frac{dS_{r,1}}{dt} = d_r u_r v_r D_{r,1} + \tau^{(FL)} w_r F_{r,1} - \left(\tau^{(SL)} + \mu\right) S_{r,1}$$

where w_r is the proportion of patients, having DR-TB and being on inappropriate first-line treatment, who are switched to second-line treatment; and $\tau^{(SL)}$ is the rate of completion of second-line TB treatment.

Temporarily dropped out of careseeking, due to missed diagnosis or loss-to-followup:

$$\frac{dB_r}{dt} = \sum_s \left[(1 - d_r u_r) D_{rs} + h_r D_{rs} + \left(1 - p^{(SL)}\right) \tau^{(SL)} S_{rs}\right] - (\gamma + \sigma + \mu_{TB}) B_s$$

Recovery following treatment completion (low relapse risk, $R^{(lo)}$):

$$\frac{dR_s^{(lo)}}{dt} = \begin{cases} \sum_r \tau^{(FL)} F_{r,0} - \left(\rho^{(lo)} + \mu + \sum_s \lambda_s\right) R_0^{(lo)} & s = 0 \\ \sum_r \tau^{(SL)} S_{r,1} - \left(\rho^{(lo)} + \mu + \sum_s \lambda_s\right) R_1^{(lo)} & s = 1 \end{cases}$$

Recovery following self-cure or incomplete treatment (high relapse risk, $R^{(hi)}$):

$$\frac{dR_s^{(hi)}}{dt} = \begin{cases} \sum_r \left[\delta_r F_{r,0} + \sigma(D_{r,0} + B_{r,0})\right] + \sigma I_0 - \left(\rho^{(hi)} + \mu + \sum_s \lambda_s\right) R_0^{(hi)} & s = 0 \\ \sum_r \left[\delta_r S_{r,1} + \sigma(D_{r,1} + B_{r,1})\right] + \sigma I_0 - \left(\rho^{(hi)} + \mu + \sum_s \lambda_s\right) R_1{}^{(hi)} & s = 1 \end{cases}$$

Force of infection (λ_s):

$$\lambda_s = \begin{cases} \beta \left[\sum_r (I_{rs} + \kappa B_{rs}) + \kappa \sum_r D_{rs}\right] & s = 0 \\ \beta_{DR} \left[\sum_r (I_{rs} + \kappa B_{rs}) + \kappa \sum_r D_{rs}\right] & s = 1 \end{cases}$$

where κ is the relative infectivity of those who have symptoms severe enough to prompt careseeking, compared to those with active TB who not sought care.

Symbol	Meaning
s	Indicator variable for strain: s = 0, 1 respectively for DS- and DR-TB
$\boldsymbol{U}$	Proportion uninfected
$\boldsymbol{L_s}$	Having *latent infection* with strain s
$\boldsymbol{I_s}$	Having *active disease* with strain s, but not yet presented for care
r	Indicator variable for provider type: r = 0 for the public sector; r = 1, 2, 3 respectively for FQ providers, LTFQ providers and chemists who are *not* engaged with the PPIA; and r = 4, 5, 6 for corresponding private providers who are engaged with the PPIA
$\boldsymbol{D_{rs}}$	Awaiting diagnosis with provider type r
$\boldsymbol{F_{rs}}$	Undergoing *first-line TB treatment* with provider type r
$\boldsymbol{S_{rs}}$	Undergoing *second-line TB treatment* with provider type r
$\boldsymbol{B_{rs}}$	Patients who have temporarily dropped out of care cascade, having visited provider type r
$\boldsymbol{R_s^{(hi)}}$	Recovered with 'high' relapse risk (treatment defaulters and spontaneous recoveries)
$\boldsymbol{R_s^{(lo)}}$	Recovered with 'low' relapse risk (following successful treatment)

Table S1. List of state variables used in the model.

2. Costing

We took the programmatic perspective for estimating costs. As shown in Table S2, we considered three different cost components: provider engagement, diagnostics, and treatment. We quantified each of these costs as follows.

Provider engagement

As described in the main text, certain providers manage a disproportionate number of TB patients. To capture the 'efficiency' of a PPIA in engaging preferentially with these providers, we recall from the model equations that p_r is the probability that a symptomatic patient visits provider type r when they seek care. If q_r is the proportion of provider type r engaged, we related these parameters as:

$$p_r = (q_r)^k,$$

allowing k to take a range of values from 0.5 (less efficient provider recruitment) to 2 (more efficient provider recruitment).

For a given level of provider engagement q_r, we estimated the corresponding costs of provider engagement using the cost estimates shown in Table S2, assuming a linear scale-up in the first three years of the intervention. We also estimated the value of p_r using the equation above, for use in the transmission model. As with all costs and benefits in this analysis, we applied discounting at a 3% annual rate.

Diagnostics

If C_{Dx} is the unit cost of each diagnostic test conducted, we estimated the total cost of diagnosis over the intervention period T as:

Total diagnostic cost = $C_{Dx} n \sum_{s,r\in[4,5,6]} \left[\int_T \left(c p_r I_s + \gamma p_r B_s \right) / (1+\epsilon)^t \ dt \right]$

where ϵ is the discounting factor, and n is the number of individuals needing to be tested in facility settings, in order to diagnose one TB case.

Treatment

If C_{FL} is the cost per patient-month of first-line treatment, we estimated the total cost of first-line treatment over the intervention period T as:

Total treatment cost = $12 \cdot C_{FL} \sum_{s,r\in[4,5,6]} \left[\int_T F_{rs} / (1+\epsilon)^t dt \right]$

where the factor 12 acts as a conversion between the unit of time in the model (years) and the unit of cost (patient-months). A corresponding expression applies for second-line TB treatment.

	Mumbai		Patna	
	FQ	LTFQ	FQ	LTFQ
**Cost per provider engaged:**				
Internal and external staff time cost	57.1*	57.1*	15.5	46.9
Provider workshop/ training cost	50.6	50.6	31.3	78.1
Monetary incentive to patients and providers	**	**	6.3	4.7
**Total**	107.7	107.7	53.0	129.7
**Cost per diagnostic test:**				
Internal and external staff time cost	24.6	0.9	5.6	†
Cost of vouchers	***	***	18.8	3.9
Cost of equipment and consumables:	19.2	2.7		
Equipment (e.g., Xpert machine)			4.4 (††)	
Consumables (e.g., Xpert cartridge)			18.1	
**Total**	43.8	3.6	27.2	3.9
**Cost per 6-month first-line treatment:**				
Internal and external staff time cost	19.1		†	
Drug cost	22.5		32.3	
Monetary incentive to patients and providers	**		9.4	
Call centre (adherence support) cost	6.6		1.9	
**Total**	48.2		43.7	

Table S2. Breakdown of cost components used in unit costs. Costs were calculated based on data collected from the NGOs in Mumbai and Patna during September 2014 to May 2015. All costs are in 2015 USD, assuming US$1= 64 Indian Rupees. Footnotes: (*) A breakdown of provider engagement costs by provider type was not available in Mumbai; we therefore assumed the same costs per FQ and LTFQ provider. In practice, the cost of LTFQ engagement is generally expected to be lower than the cost stated here for FQs; our analysis is thus conservative by tending to overestimate the overall cost of private sector engagement. (**) Patients, pharmacists, and providers in Patna were provided with cash incentives for participating in the program. No such incentives were provided in Mumbai. (***) In Patna (but not in Mumbai), all LTFQ diagnostic tests were outsourced to external laboratories through a voucher system. FQ diagnostic tests (Xpert) were divided as 90% in-house (at NGO facility which included cost of staff time, equipment, and consumables) and 10% outsourced (voucher) which yielded a weighted total of $27.2 per test. (†) In Patna, sputum/ X-ray (LTFQ) tests and first-line treatment were outsourced through a voucher system and staff time cost was not considered separately. (††) To annualize the capital cost of the Xpert machine, we considered an 15% annual depreciation value, divided by the total number of tests conducted. In Mumbai, cost of equipment and consumables were not available separately.
